# Detection of *Klebsiella pneumoniae* Carbapenem Resistance Genes by qPCR: Choosing the Right Method for Total DNA Extraction

**DOI:** 10.3390/microorganisms12071285

**Published:** 2024-06-25

**Authors:** Cecilia Heller, Iris Bachmann, Martin Spiegel, Frank T. Hufert, Gregory Dame

**Affiliations:** 1 Institute of Microbiology and Virology, Brandenburg Medical School Theodor Fontane, Universitätsplatz 1, 01968 Senftenberg, Germany; cecilia.heller@mhb-fontane.de (C.H.); iris.bachmann@mhb-fontane.de (I.B.); martin.spiegel@mhb-fontane.de (M.S.); frank.hufert@mhb-fontane.de (F.T.H.); 2 Infection Biology Unit, German Primate Center, Kellnerweg 4, 37077 Göttingen, Germany; 3 Brandenburg University of Technology Cottbus-Senftenberg, Universitätsplatz 1, 01968 Senftenberg, Germany; 4 Faculty of Health Sciences, Joint Faculty of the Brandenburg University of Technology Cottbus-Senftenberg, The Brandenburg Medical School Theodor Fontane and the University of Potsdam, Am Neuen Palais 10, House 9, 14469 Potsdam, Germany

**Keywords:** total DNA extraction, nucleic acid purification, *Klebsiella pneumoniae*, nucleic acid-based detection of carbapenem resistance, beta-lactamase detection, qPCR, synthetic stool matrix, carbapenem-resistant *Enterobacterales* (CRE)

## Abstract

Rapid and accurate detection of *Klebsiella pneumoniae* carbapenem resistance is important for infection control and targeted antibiotic therapy. PCR-based assay performance heavily depends on the quality and quantity of template DNA. Challenges arise from the necessity to isolate chromosomal and large plasmid-encoded resistance genes simultaneously from a limited number of target cells and to remove PCR inhibitors. qPCRs for the detection of *K. pneumoniae* strains carrying *bla*_OXA-48_, *bla*_NDM-1_, *bla*_KPC-2_, and *bla*_VIM-1_ carbapenemase genes were developed. We compared the performance of template DNA extracted with silica column-based methods, reversed elution systems, and lysis-only methods either from diluted culture fluid or from a synthetic stool matrix which contained PCR inhibitors typically present in stool. The synthetic stool matrix was chosen to mimic *K. pneumoniae* containing rectal swabs or stool samples in a reproducible manner. For total DNA isolated from culture fluid, resistance gene detection by qPCR was always possible, independent of the extraction method. However, when total DNA was isolated from synthetic stool matrix spiked with *K. pneumoniae*, most methods were insufficient. The best performance of template DNA was obtained with reversed elution. This highlights the importance of choosing the right DNA extraction method for consistent carbapenem resistance detection by PCR.

## 1. Introduction

Carbapenemases such as *Klebsiella pneumoniae* (*K. pneumoniae*) carbapenemase (KPC) are common in several bacteria within the *Enterobacterales* order. *K. pneumoniae*, as well as other representatives of carbapenem-resistant *Enterobacterales* (CRE), have become a critical global health challenge, causing serious implications for patient outcomes and healthcare systems [1,2]. The rate of carbapenemase-producing *Klebsiella* isolates in US hospitals has increased from <1% in 2001 to 12% in 2010 [3], while for Europe, an increasing nosocomial spread of carbapenem-resistant *K. pneumoniae* has been reported [4]. Besides KPC, carbapenemases that are becoming increasingly common in infections worldwide are Verona integron-encoded metallo-β-lactamase (VIM), imipenem-hydrolyzing metallo-β-lactamase (IMP), New Delhi metallo-β-lactamase (NDM), and oxacillinase β-lactamases (OXA, especially OXA-48) [5,6]. Infections with carbapenemase-producing *K. pneumoniae* can lead to life-threatening complications, including bloodstream infections, pneumonia, urinary tract infections, intra-abdominal infections, and even septic shock [7]. The limited treatment options available contribute to high morbidity and mortality rates, especially in immunocompromised and critically ill patients [8]. There is a clear need for immediate, rapid, and specific detection methods to detect and identify CRE in order to initiate effective antimicrobial interventions and infection control measures, as well as to improve patient outcomes [6,9,10].

Routine testing for CRE is performed with a variety of methods, including phenotypic tests such as carbapenem inactivation method-based (CIM-based) or colorimetric assays to identify the presence of carbapenemase enzymes. Nucleic acid amplification tests (NAATs) such as polymerase chain reaction (PCR), loop-mediated isothermal amplification (LAMP), isothermal recombinase polymerase amplification (RPA), or mRAP (a combination of recombinase-aided amplification and qPCR) offer high sensitivity and specificity independent of carbapenemase activity. Additionally, a short turnaround time is a significant advantage of NAATs [9,11,12,13,14,15]. Molecular techniques and especially real-time PCR assays are the reference standard for the identification of carbapenemase genes [16].

Bacteria can carry antibiotic resistance genes in their genomes or acquire resistance through horizontal gene transfer [17,18]. Therefore, multidrug resistance genes can be located on both chromosomal and plasmid DNA [18]. For carbapenemase-producing *K. pneumoniae*, large plasmids (280–300 kb) carrying resistance genes have been described [19,20]. Therefore, efficient extraction of total DNA is crucial for NAAT-based CRE testing, and the method employed directly affects quality and quantity of extracted DNA [21,22]. In addition, the isolated DNA must be free of sample components that degrade DNA or that inhibit DNA amplification in NAATs [23]. For sample material like bacterial colonies, culture fluids, or tissue samples, simple thermal lysis alone or in combination with chemical or enzymatic lysis can yield DNA pure enough for downstream applications. Furthermore, lysis-only methods provide high DNA yields if lysis of bacterial cells is complete. Lysis-only methods, however, usually fail with clinical samples which often contain NAAT inhibitors and, sometimes, components that degrade DNA, which cannot be removed by simple thermal and/or chemical lysis. Commercial nucleic acid extraction kits provide standardized protocols for isolating high-quality DNA, but may have limitations in isolating large plasmid DNA (>50 kb) or in effectively removing amplification inhibitors commonly found in clinical samples such as stool [24,25,26]. Kits that are optimized for the isolation of large plasmids use anion-exchange columns which bind the negatively charged phosphate backbone of DNA. Plasmid DNA purified by anion-exchange columns is very pure; however, the DNA yield is usually low, which is clearly a disadvantage for clinical samples that contain only a limited number of bacterial cells. Furthermore, the whole purification procedure comprises many steps, including digestion of chromosomal DNA and a final plasmid DNA precipitation and resuspension step [27]. For diagnostic purposes, anion-exchange methods are therefore usually not suitable. They are too time-consuming, do not provide satisfactory DNA yields, and cannot copurify chromosomal and plasmid DNA. Commercial kits for rapid isolation of DNA often use silica spin columns for DNA purification. After loading the lysed sample onto the column under high-salt conditions, the DNA binds via hydrophobic interactions to the silica matrix, while contaminants like PCR inhibitors do not bind and are therefore removed with the flowthrough. After a washing step, the bound DNA is eluted under low-salt conditions and is ready to use for downstream applications. However, if PCR inhibitors are bound to DNA or nonspecifically bind to the silica matrix, they will be co-eluted with the DNA. Despite this possible disadvantage, silica-based methods are routinely used for the isolation of DNA from CRE present in clinical samples when NAATs are employed for carbapenemase testing [28,29,30]. Another potential problem for silica-based methods is the often low target DNA content present in clinical samples, since they usually require an input of at least 1 µg DNA [31]. An alternative approach which was developed in recent years is the reverse elution method [32,33]. Here, the sample lysate is loaded onto a resin or onto magnetic beads capable of retaining non-nucleic acid components. The purified DNA does not bind and can be found in the flowthrough. The possible advantages of reverse elution methods are efficient removal of components that degrade DNA or inhibit downstream applications; higher recovery rates compared to silica-based methods [34], since nucleic acids are left untouched after sample lysis; and faster turnaround times due to the combination of purification and elution in one step.

In this study, we evaluated different fast DNA extraction methods with a focus on optimizing NAAT-based CRE detection. We used carbapenemase-producing *K. pneumoniae* strains and isolates as the CRE model and qPCR as the nucleic acid amplification method for the detection of carbapenem resistance genes. We evaluated seven total DNA extraction methods: two silica spin column methods, two reversed elution methods, and three lysis-only approaches. We tested the ability of the evaluated extraction methods to effectively remove amplification inhibitors present in clinical stool samples [35,36,37,38]. To mimic the bacterial load of rectal swabs [39,40], a synthetic stool matrix (Claremont BioSolutions, Upland, CA, USA) spiked with *K. pneumoniae* culture (10^5^ CFU) was used. The synthetic stool matrix contains components characteristic of stool in defined amounts, which facilitates reproducible experimental conditions [41,42,43]. *K. pneumoniae* DNA isolated with the different methods either from bacterial cultures or from spiked stool matrix was tested for the presence of the carbapenem resistance genes *bla*_OXA-48_, *bla*_NDM-1_, *bla*_KPC-2_, and *bla*_VIM-1_, as well as the *K. pneumoniae* chromosome-specific *khe* gene, by qPCR. While a combination of thermal and chemical lysis was most effective when *K. pneumoniae* DNA was isolated from bacterial cultures, reversed elution turned out to be the DNA extraction method of choice for the effective removal of amplification inhibitors present in stool samples and for the consistent and sensitive detection of carbapenemase genes.

## 2. Materials and Methods

### 2.1. qPCR

PCR primers and probes were designed using ‘*Primer3*’ software, version 4.1.0 (Estonaian node of ELIXIR - European research infrastructure for biological information, Tartu, Estonia) for the carbapenemase genes *bla*_OXA-48_ (GenBank: JN626286.1), *bla*_NDM-1_ (GenBank: KF992018.2), *bla*_KPC-2_ (GenBank: CP023480.1), and *bla*_VIM-1_ (GenBank: NG_050336.1), as well as the *K. pneumoniae* chromosome-specific *khe* gene, which encodes the hemolysin pathogenicity factor (NC_016845.1). These were provided by biomers.net (Ulm, Germany). qPCR was performed in a LightCycler 480 II (Roche Diagnostics, Mannheim, Germany) using Luna Universal Probe qPCR Mix (NEB, Ipswich, MA, USA) according to the NEB protocol in a final PCR reaction mix volume of 20 µL (20 µM each primer, 10 µM probe) with 1 µL of DNA extracts or DNA standards (n = 2). As a non-template control (NTC), 1 µL PCR-grade water (Carl Roth, Karlsruhe, Germany) was used. qPCR was performed with (1) an initial step at 95 °C for 60 s, (2) denaturation at 95 °C for 15 s and annealing/elongation at 60 °C for 30 s (45 cycles), and (3) a final hold at 40 °C for 30 s.

### 2.2. Preparation of DNA Standards

The *bla*_OXA-48_ and *bla*_NDM-1_ gene sequences (part of the GenBank sequences CP143502.1 and CP137400.1) were already available as TA-cloned inserts in plasmid pCRII (Thermo Fisher Invitrogen, Darmstadt, Germany). Sequences of *bla*_KPC-2_ (nucleotides 3326–4209 from GenBank reference sequence NZ_CP023480.1) and *bla*_VIM-1_ (nucleotides 101–901 from GenBank reference sequence NG_050336.1) were synthesized and inserted into the vectors pMA-RQ and pMA-T, respectively (Thermo Fisher Geneart, Regensburg, Germany). The integrity of the inserts was verified by Sanger sequencing (Microsynth Seqlab, Göttingen, Germany). Each plasmid was used for transformation of competent *E. coli*, strain NEB 5-alpha (NEB), according to the manufacturer’s protocol. Transformed bacteria were grown in 20 mL LB-Amp (100 mg/L) medium at 37 °C and 120 rpm in an ES-20/60 Orbital Shaker-Incubator (BioSan, Riga, Latvia) for 18 h. Plasmids were isolated using the Qiagen Midi kit (Qiagen, Hilden, Germany) according to manufacturer’s protocol. The purified plasmid DNA was linearized (*bla*_OXA-48_, *bla*_NDM-1_: *Hind* III, Thermo Scientific, Dreieich, Germany; *bla*_KPC-2_: *Nde* I Thermo Scientific; *bla*_VIM-1_: *PmI* I, NEB), and the fragments were separated by gel electrophoresis. The fragments containing the insert were purified (QIAquick Gel extraction Kit, Qiagen) and quantified (Quant-iT PicoGreen dsDNA assay, Thermo Fisher Invitrogen). A decadic dilution series from 10^7^ to 10^0^ DNA copies/µL was prepared for each resistance gene.

### 2.3. Cultivation of Klebsiella pneumoniae

The following *K. pneumoniae* strains and isolates carrying the *bla*_OXA-48_, *bla*_NDM-1_, *bla*_KPC-2_, and *bla*_VIM-1_ resistance genes were used: two clinical isolates (1: *bla*_NDM-1_; 2: *bla*_OXA-48_) and four reference strains (NRZ64515: *bla*_VIM-1_; NRZ-52799: *bla*_OXA-48_, *bla*_NDM-1_; NRZ-43730: *bla*_KPC-2_, *bla*_VIM-1_; NRZ-64650: *bla*_KPC-2_; National Reference Centre for multidrug-resistant Gram-negative bacteria (NRZ), Bochum, Germany). Bacteria were cultured overnight in LB-Amp (Lennox LB with 100 mg/L ampicillin, both Carl Roth) at 37 °C and 200 rpm. The clinical isolates and reference strains were additionally cultured on LB plates with 10, 50, and 100 mg/L meropenem (VWR International, Darmstadt, Germany) to validate resistance against carbapenems.

### 2.4. CFU/mL Estimation by OD Measurement

To estimate the CFU/mL of *K. pneumoniae* cultures by means of OD measurement, samples of reference overnight cultures of each *K. pneumoniae* strain or isolate were first diluted 1:100, and OD_600_ was determined (Helios Epsilon spectrophotometer, Thermo Scientific). Then, a dilution series of 10^−4^ to 10^−11^ for each overnight culture was plated on LB-Amp agar to determine the CFU/mL using the drop-plate method [44]. Colony counting and CFU/mL calculations were performed in triplicate for each strain. The values obtained were used to calculate calibration curves (Table S1), which were employed to estimate the CFU/mL of subsequently grown overnight cultures using OD_600_ measurements of 1:100 diluted culture samples.

### 2.5. Isolation of Klebsiella pneumoniae DNA with Different Extraction Methods

Samples of *K. pneumoniae* overnight cultures adjusted to 10^5^ CFU/200 µL were used as inputs for the different isolation methods (Table 1). To assess reproducibility, DNA extractions were carried out in three independent experiments. A negative and a process contamination control were included using PCR-grade water (Carl Roth, Karlsruhe, Germany) for each purification procedure instead of bacterial culture. Seven different total DNA extraction methods, three spin column methods (two silica columns and one reverse elution column), one plate-based method (reverse elution), and three variants of a lysis-only method (Table 1) were evaluated. For the commercially available silica spin column kits (Qiagen, Hilden and Macherey-Nagel, Düren, Germany), the reverse elution kits (BioEcho, Cologne, Germany), and the lysis-only kit (Lucigen, Middleton, WI, USA), the manufacturers’ protocols were followed, except for the BioEcho EchoLUTION Buccal Swab DNA Kit, for which the heating time for sample lysis was increased from 2 to 5 min. For DNA isolation by simple thermal lysis, the following variants were used: (i) For thermal lysis T (TL-T), 200 µL of bacterial culture was heated to 90 °C for 5 min (ThermoStat C, Eppendorf, Hamburg, Germany) prior to qPCR; (ii) for thermal lysis, P (TL-P), 1 µL of diluted bacterial culture was pipetted directly into the PCR reaction mix. Cells were lysed by an additional heating step (90 °C for 5 min, LightCycler 480 II) prior to qPCR cycles.

### 2.6. Assessment of Concentration and Quality of DNA Isolated from K. pneumoniae Strains

The concentration of isolated DNA was determined in triplicate by UV absorbance measurement at 260 nm using a Nanodrop 8000 spectrophotometer (Thermo Scientific). The instrument was blanked according to the manufacturer’s instructions. The quality of the isolated DNA was estimated with an additional absorbance measurement at 280 nm and calculation of the A260/A280 ratio.

### 2.7. Synthetic Stool Matrix as Model for Fecal Contamination

To assess the ability of the tested methods to remove compounds present in stool samples that inhibit nucleic acid amplification, 200 µL of synthetic stool mixture (Claremont BioSolutions, Upland, CA, USA) was mixed with 10^5^ CFU of pelleted bacteria of the *K. pneumoniae* strain NRZ-52799 (*bla*_OXA-48_, *bla*_NDM-1_) or the *K. pneumoniae* strain NRZ43730 (*bla*_KPC-2_, *bla*_VIM-1_). The synthetic stool matrix contained PCR-inhibitory compounds of porcine origin (e.g. bile salts, mucin, and dextran sulphate), as well as human serum albumin and human genomic DNA in defined amounts, which allowed us to maintain reproducible conditions in experimental procedures [41,42,43]. The spiked stool matrix was used for DNA isolation procedures. As negative controls, DNA isolation procedures were performed with synthetic stool matrix without spiked bacteria, and samples of the eluates were used as templates in resistance gene and *K. pneumoniae* specificity qPCR assays.

### 2.8. Processing of qPCR Data

To estimate the lower limit of detection (LOD, analytical sensitivity) of the qPCR assays, eight replicates were performed for each DNA standard dilution. The results obtained were used for probit analysis [45] using ‘*R-Studio*’ (R version 4.3.1 (2023-06-16 ucrt); RStudio version 2023.12.1 Build 402, Posit PBC, Boston, MA, USA) [46,47] to determine the lowest copy number that could be detected with 95% probability.

To compensate for the different elution volumes (*v_e_*) of the different DNA extraction methods, C_t_ values were calculated from raw threshold cycles (C_tr_) using the following equation [48]:

$$C_{t\;}{=C}_{\mathrm{tr}\;}{+\log}_{2}\;\left(\frac{v_{s}}{v_{e}}\right);\;\mathrm{with}\;\mathrm{sample}\;\mathrm{volume}\;v_{s}=200\;µL$$

Statistical analysis of PCR data was performed using Welch’s unequal variances *t*-test [49]. The full results of the statistical analysis can be found in the supplementary data (Tables S2–S4).

## 3. Results

### 3.1. qPCRs Assays for the Detection of Carbapenem Resistance Genes and the K. pneumoniae Chromosome-Specific khe Gene

Primers and probes (Table 2) were designed for the detection of the carbapenemase resistance genes *bla*_OXA-48_, *bla*_NDM-1_, *bla*_KPC-2_, and *bla*_VIM-1_ in qPCR assays. These resistance genes are not restricted to *K. pneumoniae*; they can also be found in other multi-resistant Gram-negative (MRGN) bacteria. For specificity control, primers and a probe for the *K. pneumoniae* chromosome-specific *khe* gene, which encodes the hemolysin pathogenicity factor, were designed (Table 2).

### 3.2. Analytical Sensitivity of the Newly Established qPCR Assays

**Figure 1:**
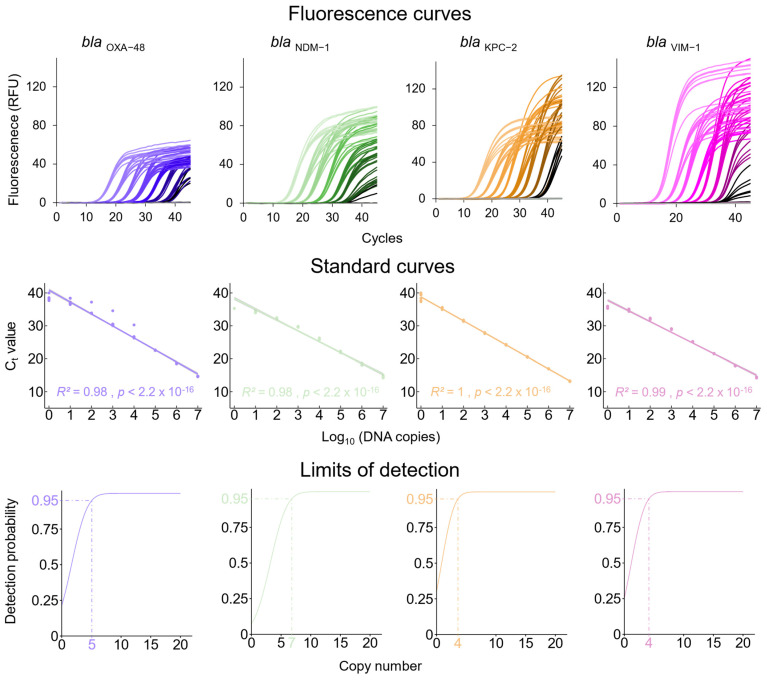
Analytical sensitivity of the qPCR assays. The newly established qPCRs for the detection of *bla*_OXA-48_, *bla*_NDM-1_, *bla*_KPC-2_, and *bla*_VIM-1_ were validated with the help of synthetic DNA standards. Dilutions ranging from 10^0^ to 10^7^ DNA copies/reaction for the DNA standards of *bla*_OXA-48_ (blue), *bla*_NDM-1_ (green), *bla*_KPC-2_ (orange), and *bla*_VIM-1_ (violet) were employed in qPCR reactions. Eight PCR reactions were performed for each DNA concentration. **Upper panel**: Fluorescence signals of the qPCR reactions. Each set of eight curves represents one DNA concentration of the decadic dilution series ranging from 10^7^ (**left**) to 10^0^ (**right**). **Middle panel**: Standard curves calculated by linear regression indicating constant performance of the qPCR assays in the tested concentration range. **Lower panel**: The limits of detection (LOD, with 95% probability), calculated by probit analysis [45], for each resistance gene assay. *bla:* beta-lactamase, NDM: New Delhi metallo-β-lactamase, OXA: oxacillinase β-lactamase, KPC: *K. pneumoniae* carbapenemase, VIM: verona integron-encoded metallo-β-lactamase, C_t_: cycle threshold, RFU: relative fluorescence unit(s).

Using the plasmid-derived DNA standards for *bla*_OXA-48_, *bla*_NDM-1_, *bla*_KPC-2_, and *bla*_VIM-1_, the linearity of the qPCR assays was determined to be in the range of 10^7^–10^0^ DNA copies/µL (Figure 1, middle panel). The limit of detection, calculated by probit analysis [46], was 4–7 DNA copies for the different resistance genes tested (Figure 1 lower panel). The LOD of the *khe* qPCR was determined to be 17 copies (Figure S1).

### 3.3. Preparation of K. pneumoniae Culture Samples for DNA Extraction

Samples of overnight cultures of the four *K. pneumoniae* strains and the two isolates were diluted 1:100, and OD_600_ was determined. Using calibration curves calculated with the help of the drop-plate method (Table S1), the CFU/mL values for the overnight cultures were estimated and adjusted to 5 × 10^5^ CFU/mL. Samples of 200 µL (corresponding to 10^5^ CFU) were used for DNA extraction.

### 3.4. Estimation of the Quantity and Quality of Total Bacterial DNA Isolated with the Different Extraction Methods

The DNA quantity and quality extracted from diluted culture samples containing 10^5^ CFU of *K. pneumoniae* were estimated spectrophotometrically at 260 nm and 280 nm. The quantity and purity (A260/A280 ratio) of the DNA varied greatly between the different methods. The highest DNA concentration was obtained with the lysis-only method, QE, which was in the range between 580 and 780 ng/µL, with low purity (A260/A280 ratio: 1.4 ± 0.02). The DNA with the highest purity (A260/A280 ratio: 1.8 ± 0.05) was achieved with the silica spin column method NS, which yielded between 51 and 103 ng/µL DNA. The reversed elution spin column method (BE) yielded 85 to 155 ng/µL DNA with high purity (A260/A280 ratio: 1.7 ± 0.04), whereas the reversed elution plate method (BV) yielded somewhat lower DNA concentrations and purity (40–140 ng/µL, A260/280 ratio: 1.5 ± 0.1). The lowest concentration and purity of DNA were obtained with the QS method (0.01–2 ng/µL, A260/280 ratio: 1.1–2.9). The concentration and purity of DNA extracted using the TL-T method could not be determined because denatured and precipitated components in the samples interfered with UV absorbance measurement. Likewise, it was not possible to determine the concentration or purity of DNA isolated from stool matrix spiked with *K. pneumoniae* culture samples by using UV/VIS spectrometry because of the human genomic DNA and other UV-active compounds which were present in the synthetic stool matrix.

### 3.5. Performance of DNA Extracted from K. pneumoniae Cultures in qPCR Assays

First, we tested DNA that was isolated from diluted bacterial cultures of the two *K. pneumoniae* reference strains and the two clinical isolates, each carrying a different resistance gene (clinical isolate 2: *bla*_OXA-48_, clinical isolate 1: *bla*_NDM-1_, NRZ-64650: *bla*_KPC-2_, NRZ-64515: *bla*_VIM-1_). To this end, 1 µL samples of total DNA isolated with the different extraction methods (Table 1) were analyzed by qPCR for the carbapenemase gene of each strain or isolate and for the *K. pneumoniae* chromosome-specific *khe* gene. For the results of the qPCR assays detecting the carbapenem resistance genes, copy numbers were calculated after adjusting C_t_ values [48] with the help of the standard curves (Figure 1, middle panel). All DNA samples of the two reference strains and the two clinical isolates tested positive for the *khe* gene, indicating the presence of chromosomal *K. pneumoniae* DNA in all extractions. Furthermore, all tested DNA isolation methods led to DNA samples which could be successfully used for detection of the carbapenemase genes in qPCR. The lowest C_t_ values and, therefore, the highest copy numbers for all four tested resistance genes were obtained with DNA isolated by the lysis-only QE method (combination of chemical and thermal lysis), showing a C_t_ value difference of 8–11 compared to the least efficient spin column method, NS. When DNA isolated with the QE-method was used as template in the resistance gene assays, in nearly all cases, Ct values were significantly lower compared to resistance gene assays with DNA isolated using the other methods (Table S2). One exception was the *bla*_VIM-1_ assay, in which DNA isolated with the reverse elution method, BV, yielded similar Ct values. Overall, DNA isolated with the silica spin column-based kits (NS, QS) achieved the highest Ct values and, therefore, the lowest copy numbers in the range of 10^0^–7 × 10^1^. With DNA isolated by the two thermal lysis-only methods (TL-T and TL-P), copy numbers in the range of 2 × 10^1^–1.86 × 10^3^ were obtained. For the two reverse elution kits (BE, BV), a range of 2.1 × 10^2^–2.16 × 10^3^ copies, and for the QE method, a range of 7 × 10^2^–6 × 10^3^ copies, were determined (Table 3).

**Figure 2:**
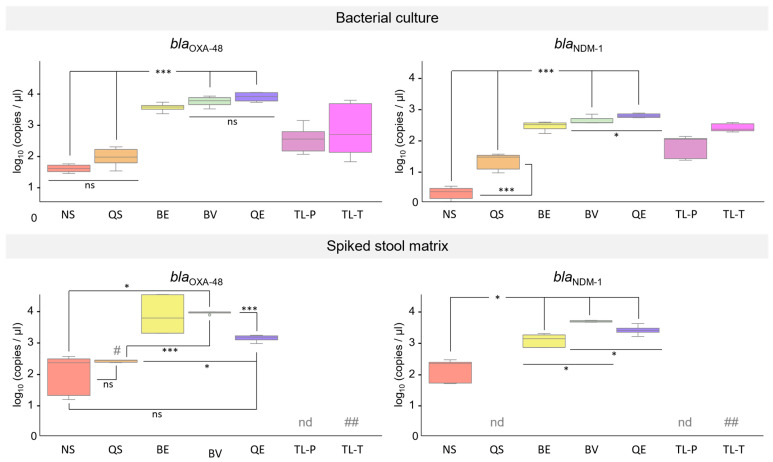
Effect of DNA extraction methods on quantitative detection of *bla*_OXA-48_ and *bla*_NDM-1_ DNA. The carbapenemase resistance genes *bla*_OXA-48_ and *bla*_NDM-1_, present in *K. pneumoniae* strain NRZ-52799, were detected by qPCR using DNA isolated with NucleoSpin DNA Stool kit (NS, red), QIAamp Fast DNA Stool Mini kit (QS, orange), EchoLUTION Buccal Swab DNA Kit (BE, yellow), EchoLUTION Viral RNA/DNA Swab Kit Plus (BV, green), Quick Extract Solution (QE, blue), Thermal lysis P (TL-P, violet), and Thermal lysis T (TL-T, pink) as templates. DNA was isolated either from bacterial culture samples (**upper panel**) or from stool matrix spiked with bacterial culture samples (**lower panel**) each containing 10^5^ CFU *K. pneumoniae* strain NRZ-52799. A sample of 1 µL of eluted DNA was used as a template in qPCR assays for the detection and quantification of *bla*_OXA-48_ and *bla*_NDM-1_. Results are shown for three independent experiments. nd: not detectable, #: inconsistent detection (only 4 of 6 PCR reactions were positive), ##: extracted DNA could not be used as a template in qPCR due to the viscosity of the samples after heat treatment. Statistical analysis was performed using Welch’s *t*-test. ns: not significant, *: *p* < 0.05; ***: *p* < 0.005.

**Figure 3:**
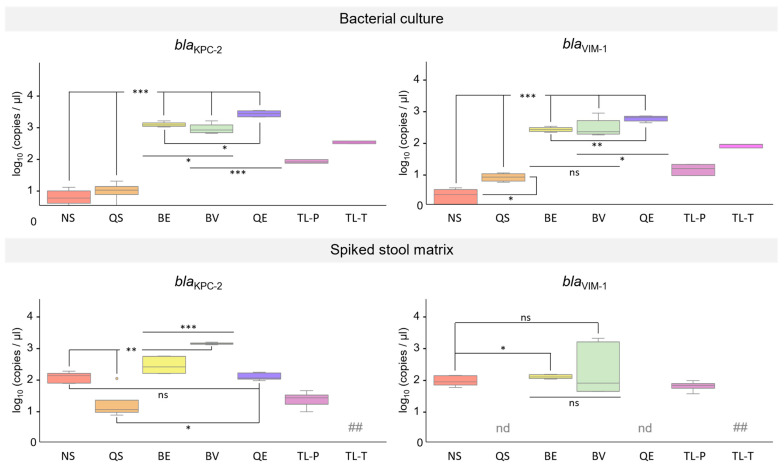
Effect of DNA extraction methods on quantitative detection of *bla*_KPC-2_ and *bla*_VIM-1_ DNA. The carbapenemase resistance genes *bla*_KPC-2_ and *bla*_VIM-1_, present in *K. pneumoniae* strain NRZ-43730, were detected by qPCR using DNA isolated with NucleoSpin DNA Stool kit (NS, red), QIAamp Fast DNA Stool Mini kit (QS, orange), EchoLUTION Buccal Swab DNA Kit (BE, yellow), EchoLUTION Viral RNA/DNA Swab Kit Plus (BV, green), Quick Extract Solution (QE, blue), Thermal lysis P (TL-P, violet), and Thermal lysis T (TL-T, pink) as template. DNA was isolated either from bacterial culture samples (**upper panel**) or from stool matrix spiked with bacterial culture samples (**lower panel**) each containing 10^5^ CFU *K. pneumoniae* strain NRZ-43730. Samples of 1 µL of eluted DNA were used as templates in qPCR assays for the detection and quantification of *bla*_KPC-2_ and *bla*_VIM-1_. Results are shown for three independent experiments. nd: not detectable, ##: extracted DNA could not be used as template in qPCR due to the viscosity of the samples after heat treatment. Statistical analysis was performed by Welch’s *t*-test. ns: not significant, *: *p* < 0.05; **: *p* < 0.01; ***: *p* < 0.005.

Next, we investigated total DNA isolated from diluted bacterial cultures of the two *K. pneumoniae* reference strains carrying two resistance genes (NRZ-52799: *bla*_OXA-48_ and *bla*_NDM-1_; NRZ-43730: *bla*_KPC-2_ and *bla*_VIM-1_). The *khe* gene was detected in all DNA samples, indicating the presence of *K. pneumoniae* chromosomal DNA in all extractions. Likewise, the detection of the resistance genes *bla*_OXA-48_ and *bla*_NDM-1_ of *K. pneumoniae* strain NRZ-52799, as well as the resistance genes *bla*_KPC-2_ and *bla*_VIM-1_ of *K. pneumoniae* strain NRZ-43730, by qPCR was successful with all DNA samples (Figure 2 and Figure 3, upper panels). Similarly to what we observed for *K. pneumoniae* strains and isolates carrying one resistance gene only, in most cases, the most efficient DNA isolation method was the QE method (combination of chemical and thermal lysis). DNA isolated with the two reversed elution methods (BE and BV) also gave rise to high copy numbers in the carbapenem resistance gene qPCR assays, followed by DNA isolated with the two thermal lysis-only methods (TL-P and TL-T) and the two silica spin column methods (NS and QS). The two reverse elution methods, BE and BV, and the lysis method, QE, yielded DNA which gave rise to significantly higher copy numbers in all four resistance gene assays in comparison to DNA isolated with the silica spin column methods, NS and QS. With DNA isolated from bacterial cultures using the lysis-only method, QE, significantly higher copy numbers were achieved in the *bla*_NDM-1_, *bla*_KPC-2_, and the *bla*_VIM-1_ resistance gene assays compared to DNA isolated with the reverse elution method, BV, whereas for the *bla*_OXA-48_ assay, no significant difference was observed (Figure 2 and Figure 3, upper panels; copy numbers expressed as log_10_ (copies/µL) and Tables S3 and S4).

### 3.6. Performance of DNA Isolated from Synthetic Stool Matrix Spiked with Cultures of K. pneumoniae Strains Carrying Two Resistance Genes in qPCR Assays

DNA samples isolated from synthetic stool matrix spiked with either 10^5^ CFU of *K. pneumoniae* strain NRZ-52799 (*bla*_OXA-48_, *bla*_NDM-1_; Figure 2) or 10^5^ CFU of *K. pneumoniae* strain NRZ-43730 (*bla*_KPC-2_, *bla*_VIM-1_; Figure 3) were used in qPCR experiments. PCR with DNA extracted from spiked stool matrix by thermal lysis with the TL-T method could not be performed due to the viscosity of the samples after heat treatment. When DNA was extracted from spiked stool matrix by thermal lysis with the TL-P method and used as a template in the carbapenem resistance gene qPCR assays, only *bla*_KPC-2_ and *bla*_VIM-1_ could be detected with low copy numbers (range of 10–100 copies, Figure 3, lower panel). With DNA isolated by the silica spin column method QS, only detection of *bla*_KPC-2_ (Figure 3, lower panel) was successful, but with low copy numbers. When DNA extracted from spiked stool matrix by the QE method (combination of chemical and thermal lysis) was used as a template in qPCR, *bla*_OXA-48_, *bla*_NDM-1_, and *bla*_KPC-2_ were successfully detected with medium (range of 100–1000 copies, *bla*_KPC-2_, Figure 3, lower panel) to high copy numbers (range of 1000–10,000 copies, *bla*_OXA-48_, *bla*_NDM-1_, Figure 2, lower panel), but the detection of *bla*_VIM-1_ failed (Figure 3, lower panel). The silica spin column method, NS, and the reverse elution methods, BE and BV, gave rise to DNA that could be successfully used for the detection of all four carbapenem resistance genes. Low to medium copy numbers were detected when DNA was used in qPCR that was isolated with the NS method, while medium to high copy numbers were detected using DNA isolated with the BE and BV methods (Figure 2 and Figure 3, lower panels respectively). For the *bla*_VIM-1_ assay, no significant difference was observed for DNA isolated with the reverse elution method, BV, compared to DNA isolated with the spin column method, NS. However, for the *bla*_OXA-48_, *bla*_NDM-1_, and *bla*_KPC-2_ assays, DNA isolated with the reverse elution method, BV, gave rise to significantly higher copy numbers compared to DNA isolated with the spin column method, NS (Figure 2 and Figure 3, lower panels; copy numbers expressed as log_10_(copies/µL) and Tables S3 and S4).

To investigate the influence of each DNA extraction method on the sensitivity of the qPCR *bla*_OXA-48_ and *bla*_NDM-1_ resistance gene assays and the assay for the *K. pneumoniae* chromosome-specific *khe* gene, we employed different extraction methods (Table 1) for the isolation of total DNA from decadic dilution series of *K. pneumoniae* strain NRZ-52799 cultures in a range from 10^5^ CFU/200 µL to 10^1^ CFU/200 µL. The diluted cultures were either used directly for DNA extraction or applied for the purpose of spiking the synthetic stool matrix, followed by DNA isolation. Samples of isolated DNAs from three independent experiments were used in duplicate as template in *bla*_OXA-48_, *bla*_NDM-1_, and *khe* qPCR assays. C_t_ values ≤ 40 were rated as positive results. Only if the qPCR assays performed in duplicate gave two positive signals in all three experiments was the detection by qPCR rated as consistent (i.e., all six qPCR reactions had to yield a positive result).

**Figure 4:**
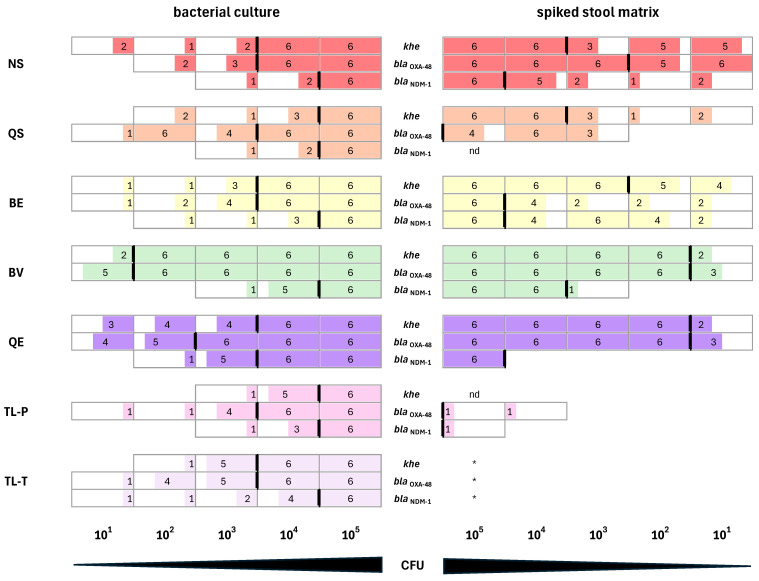
Minimal CFU input in DNA extractions, sufficient for consistent resistance gene detection. *K. pneumoniae* NRZ-52799 was diluted in the range of 10^5^ to 10^1^ CFU/200 µL of bacterial cultures (**left**), and bacterial cultures were spiked into stool matrix (**right**) and employed for the isolation of DNA with the NucleoSpin DNA Stool kit (NS, red), QIAamp Fast DNA Stool Mini kit (QS, orange), EchoLUTION Buccal Swab DNA Kit (BE, yellow), EchoLUTION Viral RNA/DNA Swab Kit Plus (BV, green), Quick Extract Solution (QE, blue), Thermal lysis P (TL-P, violet), and Thermal lysis T (TL-T, pink). Samples of 1 µL of isolated DNA were used as templates in the qPCR assays for the detection of *bla*_OXA-48_, *bla*_NDM-1_, and *khe*. Cultivation of *K. pneumoniae* NRZ-52799 and subsequent DNA extractions were performed three times in independent experiments. The isolated DNA was analyzed in duplicate in the qPCR resistance gene assays for *bla*_OXA-48_ and *bla*_NDM-1_, and in the *khe* qPCR for *K. pneumoniae* specificity control. Black bars indicate the lowest CFU input in DNA that was sufficient for consistent qPCR results (i.e., all 6 qPCR reactions were positive). nd: not detectable, *: extracted DNA could not be used as template in qPCR due to the viscosity of the samples after heat treatment.

With DNA isolated from diluted bacterial cultures as a template, the *khe*, the *bla*_OXA-48_, and the *bla*_NDM-1_ qPCR assays were always positive when 10^5^ CFUs were used as input for DNA extraction, which was in line with our previous experiments (Figure 2, upper panel). For lower CFU inputs, we observed different sensitivities depending on the DNA extraction method (Figure 4, left panel). The best result for the detection of *khe* and *bla*_OXA-48_ was achieved with DNA isolated by the reverse elution method, BV. From bacterial cultures, 10^2^ CFUs were sufficient as input for DNA isolation for consistent detection by qPCR. For the detection of *bla*_NDM-1_, the best result was achieved with DNA isolated by the lysis method, QE, with 10^4^ CFUs as input for DNA isolation.

When DNA was extracted from the spiked stool matrix, a different picture emerged (Figure 4, right panel). DNA isolated with the TL-T method could not be used for qPCR at all due to the viscosity of the samples after the heating step. Detection of the *khe K. pneumoniae* specificity control failed with DNA extracted using the TL-P lysis method. For the other extraction methods, the CFU input into the spiked stool matrix required for subsequent *khe* detection was equal (NS and BV method) or even lower (BS, BE and QE method) compared to the extractions from bacterial cultures. The best results were achieved with the BV and the QE methods. For both methods, 10^2^ CFUs were sufficient as input for DNA isolation from spiked stool matrix to consistently detect the *khe* gene using qPCR. For the detection of *bla*_OXA-48_, DNA isolated with the BV and the QE methods also yielded the best results. Again, 10^2^ CFU was sufficient as input for DNA isolation from the spiked stool matrix. Detection of *bla*_OXA-48_ was inconsistent or failed when DNA isolated by the silica spin column QS method or the lysis-only TL-P method was used as a template in the *bla*_OXA-48_ qPCR assay (Figure 4, right panel). For the detection of *bla*_NDM-1_, DNA isolated from the spiked stool matrix using the reverse elution method, BV, yielded the best results. As input for DNA isolation, 10^4^ CFUs were sufficient for consistent detection by qPCR. Detection of *bla*_NDM-1_ failed with DNA extracted from the spiked stool matrix when using the lysis method, TL-P, or was inconsistent when using the silica spin column method, QS (Figure 4, right panel). For consistent detection of *bla*_NDM-1_ using DNA isolated by the lysis method, QE, more CFU input was required for isolation from the spiked stool matrix compared to isolation from bacterial culture (10^5^ CFU instead of 10^4^ CFU). For the spin column method, NS, and the reverse elution method, BE, the same CFU input (10^5^) was required for DNA extraction from bacterial cultures and spiked stool matrix (Figure 4, right panel). Overall, when DNA isolated from the spiked stool matrix was used as a template in the *bla*_NDM-1_ and *bla*_OXA-48_ qPCR assays, the best results were achieved with DNA isolated by the reverse elution method, BV, followed by the lysis method, QE; the silica spin column method, NS; and the second reverse elution method, BE.

## 4. Discussion

Rapid identification of carbapenemase resistance in Gram-negative bacteria like *Klebsiella pneumoniae* is crucial for infection control and prevention, surveillance, and epidemiological purposes. Furthermore, it can have a significant impact upon the determination of the appropriate initial treatment for critically ill patients and upon patient outcomes in general.

There are several methods to determine carbapenem resistance. Nucleic acid-based tests like PCR, which detect resistance genes instead of enzymatic activity of the carbapenemase gene product, offer some advantages over phenotypic tests: They have a shorter turnaround time and a high sensitivity and specificity independent of the β-lactamase activity. In addition, they are better suited for high-throughput approaches because a greater number of assays can be performed in parallel with less hands-on time in comparison to phenotypic tests. Molecular diagnostics of carbapenemase resistance requires high-quality DNA that serves as a template in, e.g., PCR assays. This aspect is often overlooked, but may be crucial to avoid false negative results.

Since the DNA is usually isolated from patient samples like rectal swabs or stool, which contain only a limited number of the bacteria of interest, the chosen DNA extraction method should be very efficient. In addition, carbapenem resistance genes are often encoded on medium to large low-copy plasmids [19,20,50,51], but can also be found on the bacterial chromosome [18]. Besides that, species-specific genes used as targets, like *khe* in the case of *K. pneumoniae*, are also located on the bacterial chromosome. Therefore, an extraction method is required that works for both plasmid and bacterial chromosomal DNA. Finally, patient samples like stool or rectal swabs can contain components which are able to inhibit DNA amplification by PCR [35,36,37,38]. These inhibitors must be removed during DNA extraction to ensure consistent detection of carbapenem resistance genes by PCR.

In this work, we investigated the performance of total DNA extracted from carbapenem-resistant *K. pneumoniae* strains and isolates using seven different methods as templates in qPCR assays. To this end, we established very sensitive qPCR assays for the detection of the carbapenem resistance genes *bla*_OXA-48_, *bla*_NDM-1_, *bla*_KPC-1_, and *bla*_VIM-1_ (LOD < 10 copies) and the *K. pneumoniae* chromosome-specific *khe* gene (LOD 17 copies). When we used 10^5^ CFU *K. pneumoniae* from diluted overnight cultures as the input for DNA isolation, all tested extraction methods yielded DNA in sufficient amounts and of sufficient quality for the detection of the carbapenem resistance genes and the chromosomal *khe* gene. The purity (A260/A280 ratio) of the isolated DNA had no obvious impact on the qPCR results. We obtained very similar results for *K. pneumoniae* strains and isolates carrying only one resistance gene (Table 3) and strains carrying two resistance genes (Figure 2 and Figure 3, upper panels). In both cases (except for *K. pneumoniae* NRZ-64515 *bla*_VIM-1_), the best results were achieved with DNA isolated by the QE method, a combination of chemical and thermal lysis, which also gave rise to the highest DNA yield. But even DNA extraction by simple thermal lysis, either as separate step prior to PCR (TL-T method) or as first step of PCR (TL-P method), was sufficient to consistently detect the resistance genes and the *khe* gene. To our surprise, simple thermal lysis worked better than DNA isolation using silica spin columns. DNA isolation from bacterial cultures with the silica spin column methods NS and QS resulted in the lowest DNA yields and the lowest copy numbers in the carbapenem resistance gene qPCR assays. DNA extraction with the reverse elution methods BE and BV was much more efficient in terms of DNA yield and led to much higher copy numbers in the carbapenem resistance gene qPCR assays, but had no advantage over DNA isolated by the lysis-only QE method. In summary, when diluted bacterial cultures were used as input for the isolation of DNA, lysis-only methods were entirely sufficient for the subsequent detection of carbapenem resistance in qPCR assays.

Clinical samples, like rectal swabs, which are used in carbapenem resistance testing contain compounds which can inhibit PCR [2,52]. To investigate whether the DNA extraction methods were capable of removing these inhibitors, we spiked a synthetic stool matrix with 10^5^ CFU of the *K. pneumoniae* strains NRZ-52799 or NRZ-43730. The synthetic stool matrix is a mixture of compounds mimicking stool, including PCR inhibitors present in relevant amounts in actual stool, like bile salts, mucin, serum albumin, and dextran sulfate [52,53,54]. In contrast to an actual stool specimen, in synthetic stool matrix, these compounds are present in defined amounts. This enabled us to investigate the performance of isolated DNA under reproducible conditions.

The spiked stool matrix samples were used as inputs for the different DNA extraction methods. It turned out that simple thermal lysis resulted in DNA samples which either could not be used at all (due to high viscosity, TL-T method) or which could only be amplified in two of the resistance gene assays (*bla*_KPC-2_ and *bla*_VIM-1_), but not in the *khe* assay (TL-P method). DNA isolated with the QE method (combination of chemical and thermal lysis) performed very well in the *khe* assay (Figure 4, right panel) and could also be used for the detection of the resistance genes *bla*_OXA-48_, *bla*_NDM-1_, and *bla*_KPC-2_, but not for the detection of *bla*_VIM-1_. The failure of the *bla*_VIM-1_ assay using DNA isolated with the QE method as a template was unexpected, since DNA isolated by the thermal lysis-only method, TL-P, was successfully amplified. Presumably, components of the QE lysis buffer might negatively affect the performance of the *bla*_VIM-1_ qPCR. The QE method is not optimized for DNA isolation from fecal samples. Nevertheless, DNA isolated from the spiked stool matrix using the QE method could be successfully used for the detection of the resistance genes *bla*_OXA-48_, *bla*_NDM-1_, and *bla*_KPC-2_. Since the QE method has a very simple and fast protocol, it could be the method of choice for future point-of-need carbapenemase gene assays provided that a further optimization of the lysis buffer can be achieved.

DNA isolated from the spiked stool matrix with the two silica spin column methods, QS and NS, behaved very differently in the qPCR assays. With DNA isolated by the NS method, detection of all four resistance genes, as well as the *khe* gene, was successful. In contrast, with DNA isolated by the QS method, detection of only the *khe* and the *bla*_KPC-2_ genes was possible, whereas detection of the *bla*_OXA-48_ gene was inconsistent (Figure 2, lower panel and Figure 4, right panel) and detection of *bla*_NDM-1_ and *bla*_VIM-1_ failed (Figure 2 and Figure 3, lower panels). Both silica spin column methods are optimized for the isolation of human and bacterial chromosomal DNA from stool samples, and both kits contain buffers for the removal of PCR inhibitors during sample lysis [55,56]. The two methods differ, however, in the way in which lysis is performed. While in both methods, the resuspended samples are incubated at 70 °C for thermal lysis, only the NS method combines heating with a mechanical lysis step, which is performed by vigorous shaking of the sample in the presence of ceramic beads. It is likely that the better DNA yield of the NS method compared to the QS method that we observed after total DNA isolation from *K. pneumoniae* cultures can be attributed to the additional bead-based mechanical lysis. It has been demonstrated that bead beating is very efficient in breaking down bacterial cells and releasing DNA [57,58]. Furthermore, an additional step for removing inhibitors was only present in the NS method. Before the transfer onto the silica spin column for DNA binding, the lysed sample was filtered through a clean-up column, while in the QS method, the lysate was directly transferred onto the silica spin column. For the resistance gene qPCR assays, an additional PCR inhibitor removal step by means of the filter column seemed to be required for the consistent amplification of DNA isolated from the spiked stool matrix. Apart from the silica spin column NS method, both reverse elution methods, BE and BV, were suitable for isolating DNA from the spiked stool matrix for usage in *K. pneumoniae* resistance gene testing by qPCR. Both reverse elution methods make use of a purification matrix that binds impurities and cellular debris while DNA passes through without interaction. They differ, however, in the composition of the lysis buffer and the lysis procedure. With the BE method, lysis is performed in an enzyme-free buffer for 5 min at 80 °C, whereas with the BV method, lysis is performed for 10 min at 95 °C. For the detection of *bla*_OXA-48_, *bla*_NDM-1_, and *bla*_KPC-2_ using DNA isolated from spiked stool matrix as a template, the lysis procedure of the BV method seemed to be more effective than the lysis procedure of the BE method. We could detect higher copy numbers of these resistance genes when using DNA extracted with the BV method from stool matrix spiked with 10^5^ CFU *K. pneumoniae* (Figure 2 and Figure 3, lower panels). In the case of *bla*_OXA-48_ and *bla*_NDM-1_, we additionally determined the minimum CFU input required for DNA isolation in order to consistently detect the resistance genes. While 10^2^ CFUs for *bla*_OXA-48_ and 10^4^ CFUs for *bla*_NDM-1_ were sufficient when using the BV method for DNA extraction, 10^5^ CFUs were required for the BE method. Likewise, for the detection of the *K. pneumoniae* chromosome-specific *khe* gene, 10^2^ CFUs were sufficient as input for DNA isolation by the BV method, whereas 10^5^ CFUs were required for DNA isolation by the BE method. Interestingly, for *bla*_VIM-1_, higher copy numbers could be detected when using DNA isolated by the BE method from bacterial cultures (Table 3 and Figure 3, upper panel) or from spiked stool samples than with DNA isolated by the BV method (Figure 3, lower panel). We have no concise explanation for this phenomenon, although it is tempting to speculate that a component of the BV lysis buffer might negatively affect the performance of the *bla*_VIM-1_ qPCR assay. Nevertheless, when using 10^5^ CFUs *K. pneumoniae* as input, which is a typical amount present in clinical samples [39,40], both reverse elution methods provided DNA in sufficient amounts and of sufficient quality for consistent resistance gene detection by qPCR. Interestingly, both reverse elution systems are optimized for the isolation of viral nucleic acids and are not intended for the isolation of total bacterial DNA. Despite this fact, the lysis procedure, especially that of the BV method, efficiently released total DNA from bacterial cells, and both reverse elution methods yielded bacterial DNA free of PCR inhibitors. Thus, they are valid alternatives to silica spin column methods like the NS method. It should also be noted that the reverse elution methods require considerably less time (15–30 min) compared to the silica spin column methods due to their simplified workflow. In the future, reverse elution methods might, therefore, be the DNA isolation methods of choice for rapid carbapenem resistance detection, especially with the help of novel point-of-need assays. Furthermore, for the reverse elution method, BV, 10-fold to 100-fold less bacterial input was required compared to the silica spin column method, NS, to provide enough DNA for consistent resistance gene detection from the spiked stool matrix. This opens up the possibility to detect infections with carbapenem-resistant *K. pneumoniae* earlier than is currently possible. In a clinical setting, this will help to improve the treatment outcomes of infected patients, since antibiotic therapy can be already initiated when bacterial load is low and the pathogen is, therefore, easier to eradicate. Apart from improved outcomes for the individual patient, this might also help to reduce the spread of carbapenem-resistant bacteria and thereby help to reduce the number of nosocomial infections.

## 5. Conclusions

Nucleic acid amplification methods like qPCR offer high sensitivity and high specificity for carbapenem resistance gene detection. For this aim, template DNA in sufficient concentrations and of sufficient purity is required. Despite the complex composition and the limited pathogen content of clinical samples, the chosen DNA extraction method must provide efficient lysis and removal of PCR inhibitors, as well as a high total DNA yield.

Both established silica spin column methods and newer reverse elution methods can fulfill these requirements, but reverse elution methods offer some advantages. On the one hand, they have a shorter turnaround time due to the combination of purification and elution in one step. On the other hand, they deliver higher DNA yields, since they leave the DNA untouched after lysis while inhibitors are efficiently removed by binding to a matrix. Therefore, consistent detection of *K. pneumoniae* carbapenem resistance genes by qPCR can be improved by using template DNA isolated with reverse elution methods.

## Figures and Tables

**Table 1:** Compared DNA extraction methods. The DNA extraction methods whose characteristics are compared in this study are divided into column-based, plate-based, and lysis-only methods. The hands-on time was estimated for 3 samples, and the list prices per reaction were calculated using online prices (from August 2023, Germany).

Extraction Method (Abbreviation)			Extraction Principle	Intended Use	Elution Volume (µL)	Hands on Time	List Price per Reaction (EUR)
column-based	NucleoSpin DNA Stool kit, Macherey-Nagel	(NS)	chemical lysis, heating, and mechanical lysis (bead beating); clean-up column; silica spin column (for binding of DNA and subsequent elution)	Isolation of human and bacterial DNA from stool samples	50	≈1 h	5.52
	QIAamp Fast DNA Stool Mini kit, Qiagen	(QS)	chemical lysis, heating; silica spin column (for binding of DNA and subsequent elution)	Isolation of human and bacterial DNA from stool samples	200	≈1 h	7.22
	EchoLUTION Buccal Swab DNA Kit, BioEcho	(BE)	chemical lysis, heating; reverse elution (DNA in flow-through); spin column format	Isolation of viral DNA from buccal swab samples	100	≈30 min	3.30
plate-based	EchoLUTION Viral RNA/DNA Swab Kit Plus, BioEcho	(BV)	chemical and enzymatic lysis, heating; reverse elution (DNA in flow-through); plate format	Isolation of viral DNA/RNA from naso- and oropharyngeal swabs or stool samples	90	≈15 min	4.62
lysis-only	Quick Extract Solution, Lucigen	(QE)	heating, chemical lysis	Isolation of pro- and eukaryotic DNA from bacterial colonies and eucaryotic tissues	400	9 min	2.84
	Thermal lysis P	(TL-P)	heating (as first step of qPCR)	Isolation of prokaryotic DNA from bacterial colonies	200	6 min	-
	Thermal lysis T	(TL-T)	heating (as separate step in thermoblock)	Isolation of prokaryotic DNA from bacterial colonies	200	6 min	-

**Table 2:** qPCR primer and probes. The oligonucleotide primer and probe sequences of the qPCR assays for the detection of *bla*_OXA-48_, *bla*_NDM-1_, *bla*_KPC-2_, *bla*_VIM-1_, and *khe* are shown. The fluorescence probes are labeled with fluorescein (6-FAM) and quenched with either BMN-Q535 or BHQ1.

Oligonucleotide		Sequence (5′ → 3′)
*bla*_OXA-48_	forward primer	GATGGACAGACGCGCGATA
	reverse primer	ACTGAATATTTCATCGCGGTGAT
	probe	(6-FAM)-CGCCACTT(BMN-Q535)GGAATCGCGATCATAATC-(BMN-Q535)
*bla*_NDM-1_	forward primer	CGTGCTGGTGGTCGATACC
	reverse primer	CCTGCTTGATCCAGTTGAGGAT
	probe	(6-FAM)-CCTGGACC(BMN-Q535)GATGACCAGACCGC-(BMN-Q535)
*bla*_KPC-2_	forward primer	CGCGGAACCATTCGCTAA
	reverse primer	CGGTATCCATCGCGTACACA
	probe	(6-FAM)-CTCGAACCA(BMN-Q535)GGACTTTGGCGGCTCC-(BMN-Q535)
*bla*_VIM-1_	forward primer	CGCTTCGGTCCAGTAGAGCTCT
	reverse primer	CCACCGTATAGCACGTTCGCTG
	probe	(6-FAM)-TCCTGGTG(BMN-Q535)CTGCGCATTCGACCGACA-(BMN-Q535)
*khe*	forward primer	GATGAAACGACCTGATTGCATTC
	reverse primer	CCGGGCTGTCGGGATAAG
	probe	(6-FAM)-CGCGAACTGGAAGGGCCCG-(BHQ1)

*bla*: beta-lactamase, NDM: New Delhi metallo-β-lactamase, OXA: oxacillinase β-lactamase, KPC: *K. pneumoniae* carbapenemase, VIM: verona integron-encoded metallo-β-lactamase, *khe*: *K. pneumoniae* hemolysin, 6-Fam: 6-Carboxyfluorescein (absorption maximum: 495 nm), BMN-Q535: ‘biomers.net’ quencher (absorption maximum: 535 nm), BHQ1: Black Hole Quencher 1 (absorption maximum: 534 nm).

**Table 3:** qPCR performance of extracted *K. pneumoniae* DNA. DNA was extracted from bacterial cultures of two clinical isolates, 2 (*bla*_OXA-48_) and 1 (*bla*_NDM-1_), and two *K. pneumoniae* reference strains, NRZ-64650 (*bla*_KPC-2_) and NRZ-64515 (*bla*_VIM-1_), with the indicated extraction methods. Samples of 1 µL DNA were employed as templates in qPCR assays detecting carbapenemase genes. Shown are the adjusted mean C_t_ values (±standard deviations) [48] and corresponding averaged copy numbers of three independent experiments.

Extraction Method	Targeted Gene	C_t_ Value	Copy Number
NS	*bla*_OXA-48_	35.8 (±0.3)	10
	*bla*_NDM-1_	36.1 (±0.5)	3
	*bla*_KPC-2_	38.4 (±0.6)	1
	*bla*_VIM-1_	35.9 (±0.7)	3
QS	*bla*_OXA-48_	33.6 (±1.5)	70
	*bla*_NDM-1_	33.8 (±0.7)	20
	*bla*_KPC-2_	36.9 (±0.6)	3
	*bla*_VIM-1_	35.0 (±0.4)	6
BE	*bla*_OXA-48_	31.0 (±1.8)	380
	*bla*_NDM-1_	30.2 (±0.8)	210
	*bla*_KPC-2_	29.2 (±0.3)	420
	*bla*_VIM-1_	28.0 (±0.3)	850
BV	*bla*_OXA-48_	28.5 (±1.8)	2160
	*bla*_NDM-1_	29.0 (±0.8)	520
	*bla*_KPC-2_	29.0 (±1.4)	520
	*bla*_VIM-1_	28.3 (±0.9)	820
QE	*bla*_OXA-48_	26.7 (±0.7)	6010
	*bla*_NDM-1_	28.7 (±0.6)	1340
	*bla*_KPC-2_	28.1 (±0.2)	870
	*bla*_VIM-1_	28.4 (±0.5)	700
TL-T	*bla*_OXA-48_	28.8 (±0.1)	1560
	*bla*_NDM-1_	30.8 (±0.5)	140
	*bla*_KPC-2_	32.1 (±0.7)	70
	*bla*_VIM-1_	31.5 (± 0.5)	80
TL-P	*bla*_OXA-48_	28.5 (±0.4)	1860
	*bla*_NDM-1_	33.1 (±0.3)	20
	*bla*_KPC-2_	34.3 (±3.7)	80
	*bla*_VIM-1_	33.4 (±0.8)	20

*bla*: beta-lactamase, NDM: New Delhi metallo-β-lactamase, OXA: oxacillinase β-lactamase, KPC: *K. pneumoniae* carbapenemase, VIM: verona integron-encoded metallo-β-lactamase, C_t_: cycle threshold.

## Data Availability

The raw data supporting the conclusions of this article will be made available by the authors upon request.

## Supplementary Materials

The following supporting information can be downloaded at: https://www.mdpi.com/article/10.3390/microorganisms12071285/s1, Table S1: Calibration curves for estimation of CFU/mL of *K. pneumoniae*; Figure S1: Evaluation of *khe* qPCR assays. Tables S2–S4: Statistical analysis of resistance gene assays using *K. pneumoniae* DNA extracted with different methods.

## Abbreviations

A260/A280 ratio	absorbance ratio 260 nm/280 nm
BE	EchoLUTION Buccal Swab DNA Kit, BioEcho
BV	EchoLUTION Viral RNA/DNA Swab Kit Plus, BioEcho
*bla*	beta-lactamase/β-lactamase
CFU	colony-forming unit(s)
CIM	carbapenem inactivation method
CRE	carbapenem-resistant *Enterobacterales*
C_t_	cycle threshold
C_tr_	raw cycle threshold
IMP	imipenem-hydrolyzing metallo-β-lactamase
*khe*	*K. pneumoniae* hemolysin
KPC	*K. pneumoniae* carbapenemase
*K. pneumoniae*	*Klebsiella pneumoniae*
LAMP	loop-mediated isothermal amplification
LOD	limit of detection
MRGN	multi-resistant Gram-negative
NAAT	Nucleic acid amplification test
NDM	New Delhi metallo-β-lactamase
NRZ	National Reference Centre for multidrug-resistant Gram-negative bacteria, Germany
NS	NucleoSpin DNA Stool kit, Macherey-Nagel
NTC	non-template control
OD_600_	optical density 600 nm
OXA	oxacillinase β-lactamase
PCR	polymerase chain reaction
QE	Quick Extract solution, Lucigen
qPCR	quantitative polymerase chain reaction
QS	QIAamp Fast DNA Stool Mini kit, Qiagen
RFU	relative fluorescence unit(s)
RPA	recombinase polymerase amplification
rpm	revolutions per minute
SD	standard deviation
TL-P	thermal lysis PCR, direct PCR
TL-T	thermal lysis thermoblock
UV/VIS	ultraviolet/visible
v_e_	elution/extraction volume
VIM	verona integron-encoded metallo-β-lactamase
v_s_	sample volume
