# Supplementary material for: Detection of Klebsiella pneumoniae Carbapenem Resistance Genes by qPCR: Choosing the Right Method for Total DNA Extraction
Source: Microorganisms. 2024 Jun 25;12(7):1285. doi: 10.3390/microorganisms12071285 (PMC11278521; doi:10.3390/microorganisms12071285)
Supplement: Supplementary file 1 [file microorganisms-12-01285-s001.zip › microorganisms-3038052-supplementary.pdf]

Supplementary Information

Detection of *Klebsiella pneumoniae* carbapenem resistance genes by qPCR: choosing the right method for total DNA extraction

Cecilia Heller[1, †], Iris Bachmann[1, †], Martin Spiegel[1, 2], Frank T. Hufert[1, 3, 4] and Gregory Dame[1, 4 *]

[1] Institute of Microbiology and Virology, Brandenburg Medical School Theodor Fontane, Universitätsplatz 1, 01968 Senftenberg, Germany; cecilia.heller@mhb-fontane.de (C.H.); iris.bachmann@mhb-fontane.de (I.B.); martin.spiegel@mhb-fontane.de (M.S.); frank.hufert@mhb-fontane.de (F.T.H.); gregory.dame@mhb-fontane.de (G.D.)

[2] Infection Biology Unit, German Primate Center, Kellnerweg 4, 37077 Göttingen, Germany

[3] Brandenburg University of Technology Cottbus-Senftenberg, Universitätsplatz 1, 01968 Senftenberg, Germany

[4] Faculty of Health Sciences, Joint Faculty of the Brandenburg University of Technology Cottbus-Senftenberg, The Brandenburg Medical School Theodor Fontane and the University of Potsdam Am Neuen Palais 10, House 9, 14469 Potsdam, Germany

* Correspondence: gregory.dame@mhb-fontane.de; Tel.: +49-3573-85901

† These authors contributed equally to this work.

Table S1. Calibration curves determined by drop plate method for estimation of CFU/ml of *K. pneumoniae* cultures by OD_{600} measurement of 1:100 diluted overnight cultures.

K. pneumoniae **strain, resistance gene(s)**	**Equation for calculation of CFU/ml (y) from OD_{600} (x)**	**CFU/ml (± SD) corresponding to an OD_{600} of 0.04**
K. pneumoniae (clinical isolate), bla_{OXA-48}	$y = 5 \cdot 10^8x + 7 \cdot 10^{-8}$	$2.0 (\pm 0.4) \cdot 10^7$
K. pneumoniae (clinical isolate), bla_{NDM-1}	$y = 7 \cdot 10^8x$	$2.8 (\pm 0.3) \cdot 10^7$
K. pneumoniae (NRZ-52799), bla_{OXA-48} and bla_{NDM-1}	$y = 1 \cdot 10^9x$	$4.0 (\pm 0.4) \cdot 10^7$
K. pneumoniae (NRZ-64650), bla_{KPC-2}	$y = 1 \cdot 10^9x$	$4.0 (\pm 1.0) \cdot 10^7$
K. pneumoniae (NRZ-64515), bla_{VIM-1}	$y = 1 \cdot 10^9x + 1 \cdot 10^{-7}$	$4.0 (\pm 0.8) \cdot 10^7$
K. pneumoniae (NRZ-43730), bla_{KPC-2} and bla_{VIM-1}	$y = 1 \cdot 10^9x$	$4.0 (\pm 1.2) \cdot 10^7$

SD: standard deviation, CFU: colony forming unit(s), OD_{600}: optical density 600 nm, *bla:* beta-lactamase, NDM: New Delhi metallo-β-lactamase, OXA: oxacillinase β-lactamase, KPC: *K. pneumoniae* carbapenemase, VIM: verona integron-encoded metallo-β-lactamase, NRZ: National Reference Centre for multidrug-resistant Gram-negative bacteria, Germany

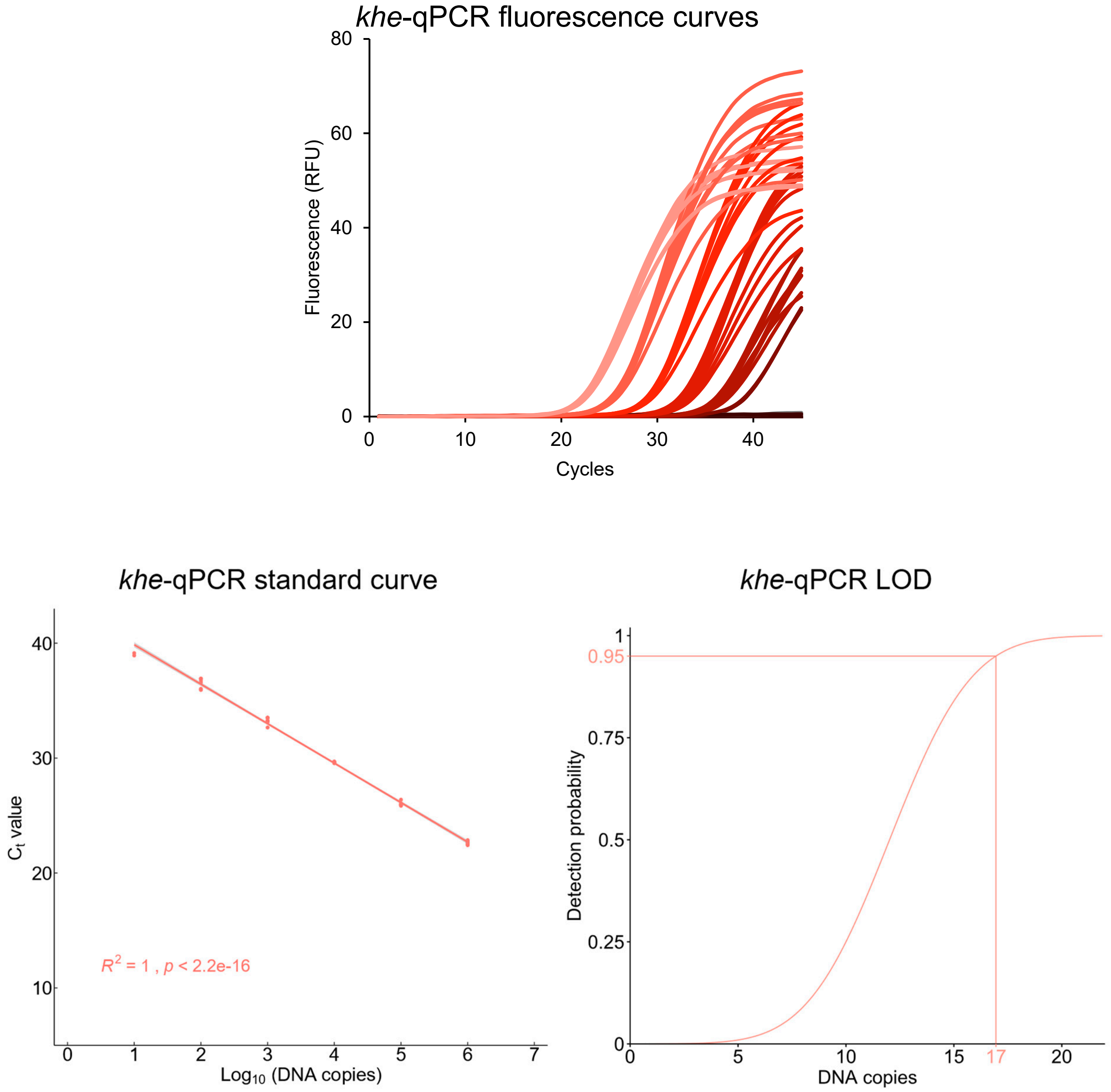

Figure S1. Analytical sensitivity of *khe* qPCR assay. The newly established qPCR for the detection of *khe* was validated with the help of a synthetic DNA standard. Dilutions ranging from 10^0 to 10^6 DNA copies/reaction for the DNA standards of *khe* were employed in qPCR reactions. Eight PCR reactions were performed for each DNA concentration. Upper panel: Fluorescence signals of the qPCR reactions. Each set of eight curves represents one DNA concentration of the decadic dilution series ranging from 10^6 (left) down to 10^0 (right). Lower panel: Left: Standard curves calculated by linear regression indicating constant performance of the *khe* qPCR assay in the tested concentration range. Right: The limit of detection (LOD, with 95 % probability), calculated by probit analysis [46] for the *khe* gene assay. *khe: Klebsiella pneumoniae* hemolysin C_t: cycle threshold, RFU: relative fluorescence unit(s).

Table S2. Statistical analysis based on Ct values of resistance gene assays using *K. pneumoniae* DNA extracted with different methods. DNA was extracted from bacterial cultures (10^5 CFU) of the two clinical isolates 2 (*bla*$_{\text{OXA-48}}$) and 1 (*bla*$_{\text{NDM-1}}$) and the two *K. pneumoniae* reference strains NRZ-64650 (*bla*$_{\text{KPC-2}}$) and NRZ-64515 (*bla*$_{\text{VIM-1}}$) with the indicated methods and used as template in the indicated resistance gene assays. Achieved Ct values were analyzed by Welch's *t*-test. NS: NucleoSpin DNA Stool kit, QS: QIAamp Fast DNA Stool Mini kit BE: EchoLUTION Buccal Swab DNA Kit, BV: EchoLUTION Viral RNA/DNA Swab Kit Plus, QE: Lucigen Quick Extract Solution , TL-P: Thermal lysis P, TL-T: Thermal lysis TL-T, pink). Statistical significance is indicated in the upper areas as follows: ns: not significant, *: $p < 0.05$; **: $p < 0.01$; ***: $p < 0.005$. Outcome in the lower areas is indicated as follows: <, Ct value achieved with DNA isolated with the method indicated in the first column is significantly lower than the Ct value achieved with DNA isolated with the indicated method in the first row. >, Ct value achieved with DNA isolated with the method indicated in the first column is significantly higher than the Ct value achieved with DNA isolated with the indicated method in the first row. =, no significant difference in Ct values.

Bacterial culture

***bla*$_{\text{OXA-48}}$**

	NS	QS	BE	BV	QE	TL-P	TL-T
NS		ns	***	***	***	***	***
QS	=		ns	**	*	*	*
BE	<	=		***	***	***	***
BV	<	<	<		**	ns	ns
QE	<	<	<	<		***	***
TL-P	<	<	<	=	>		ns
TL-T	<	<	<	=	>	=	

***bla*$_{\text{NDM-1}}$**

	NS	QS	BE	BV	QE	TL-P	TL-T
NS		***	***	***	***	***	***
QS	<		***	***	***	ns	***
BE	<	<		***	***	***	ns
BV	<	<	<		***	***	**
QE	<	<	<	<		***	***
TL-P	<	=	>	>	>		**
Tl-T	<	<	=	>	>	<	

***bla*$_{\text{KPC-2}}$**

	NS	QS	BE	BV	QE	TL-P	TL-T
NS		***	***	***	***	ns	***
QS	<		***	***	***	ns	***
BE	<	<		ns	***	ns	*
BV	<	<	=		*	ns	***
QE	<	<	<	<		ns	*
TL-P	=	=	=	=	=		ns
TL-T	<	<	>	>	>	=	

***bla*$_{\text{VIM-1}}$**

	NS	QS	BE	BV	QE	TL-P	TL-T
NS		*	***	***	***	*	***
QS	<		***	***	***	ns	***
BE	<	<		ns	ns	**	***
BV	<	<	=		ns	***	***
QE	<	<	=	=		***	***
TL-P	<	=	=	>	>		*
Tl-T	<	<	>	>	>	<	

bla: beta-lactamase, NDM: New Delhi metallo-β-lactamase, OXA: oxacillinase β-lactamase, KPC: *K. pneumoniae* carbapenemase, VIM: verona integron-encoded metallo-β-lactamase, NRZ: National Reference Centre for multidrug-resistant Gram-negative bacteria, Germany

Table S3. Statistical analysis based on log$_{10}$(copies/µl) values of *bla*$_{\text{OXA-48}}$ and *bla*$_{\text{NDM-1}}$ resistance gene assays using *K. pneumoniae* DNA extracted with different methods. DNA was extracted from bacterial culture samples (upper panel) and from stool matrix spiked with bacterial culture samples (lower panel) each containing 10^5 CFU *K. pneumoniae* strain NRZ-52799 with the indicated methods and used as template in the indicated resistance gene assays. Achieved log$_{10}$(copies/µl) values were analyzed by Welch's *t*-test. NS: NucleoSpin DNA Stool kit, QS: QIAamp Fast DNA Stool Mini kit BE: EchoLUTION Buccal Swab DNA Kit, BV: EchoLUTION Viral RNA/DNA Swab Kit Plus, QE: Lucigen Quick Extract Solution, TL-P: Thermal lysis P, TL-T: Thermal lysis TL-T, pink). Statistical significance is indicated in the upper areas as follows: ns: not significant, *: $p < 0.05$; **: $p < 0.01$; ***: $p < 0.005$. Outcome in the lower areas is indicated as follows: <, log$_{10}$(copies/µl) value achieved with DNA isolated with the method indicated in the first column is significantly lower than the log$_{10}$(copies/µl) value achieved with DNA isolated with the indicated method in the first row. >, log$_{10}$(copies/µl) value achieved with DNA isolated with the method indicated in the first column is significantly higher than the log$_{10}$(copies/µl) value achieved with DNA isolated with the indicated method in the first row. =, no significant difference in log$_{10}$(copies/µl) values. Fields highlighted in light grey indicate that one or two data sets were not available for analysis.

Bacterial culture

bla$_{\text{OXA-48}}$

	NS	QS	BE	BV	QE	TL-P	TL-T
NS		ns	***	***	***	***	***
QS	=		**	***	***	*	*
BE	>	>		**	*	***	*
BV	>	>	>		ns	***	*
QE	>	>	>	=		***	**
TL-P	>	>	<	<	<		ns
TL-T	>	>	<	<	<	=	

bla$_{\text{NDM-1}}$

	NS	QS	BE	BV	QE	TL-P	TL-T
NS		***	***	***	***	***	***
QS	>		***	***	***	*	***
BE	>	>		*	***	***	ns
BV	>	>	>		*	***	ns
QE	>	>	>	>		***	***
TL-P	>	>	<	<	<		***
TL-T	>	>	=	=	<	>	

Spiked stool matrix

bla$_{\text{OXA-48}}$

	NS	QS	BE	BV	QE	TL-P	TL-T
NS		ns	*	*	ns		
QS	=		ns	***	*		
BE	>	=		ns	ns		
BV	>	>	=		***		
QE	=	>	=	<			
TL-P							
TL-T							

bla$_{\text{NDM-1}}$

	NS	QS	BE	BV	QE	TL-P	TL-T
NS			*	*	*		
QS							
BE	>			*	ns		
BV	>		>		*		
QE	>		=	>			
TL-P							
TL-T							

bla: beta-lactamase, NDM: New Delhi metallo-β-lactamase, OXA: oxacillinase β-lactamase, KPC: *K. pneumoniae* carbapenemase, VIM: verona integron-encoded metallo-β-lactamase, NRZ: National Reference Centre for multidrug-resistant Gram-negative bacteria, Germany

Table S4. Statistical analysis based on log10(copies/µl) values of *bla*KPC-2 and *bla*VIM-1 resistance gene assays using *K. pneumoniae* DNA extracted with different methods. DNA was extracted from bacterial culture samples (upper panel) and from stool matrix spiked with bacterial culture samples (lower panel) each containing 10^5 CFU *K. pneumoniae* strain NRZ-43730 with the indicated methods and used as template in the indicated resistance gene assays. Achieved log10(copies/µl) values were analyzed by Welch's *t*-test. NS: NucleoSpin DNA Stool kit, QS: QIAamp Fast DNA Stool Mini kit BE: EchoLUTION Buccal Swab DNA Kit, BV: EchoLUTION Viral RNA/DNA Swab Kit Plus, QE: Lucigen Quick Extract Solution, TL-P: Thermal lysis P, TL-T: Thermal lysis TL-T, pink). Statistical significance is indicated in the upper areas as follows: ns: not significant, *: $p < 0.05$; **: $p < 0.01$; ***: $p < 0.005$. Outcome in the lower areas is indicated as follows: <, log10(copies/µl) value achieved with DNA isolated with the method indicated in the first column is significantly lower than the log10(copies/µl) value achieved with DNA isolated with the indicated method in the first row. >, log10(copies/µl) value achieved with DNA isolated with the method indicated in the first column is significantly higher than the log10(copies/µl) value achieved with DNA isolated with the indicated method in the first row. =, no significant difference in log10(copies/µl) values. Fields highlighted in light grey indicate that one or two data sets were not available for analysis.

Bacterial culture

*bla*KPC-2

	NS	QS	BE	BV	QE	TL-P	TL-T
NS		ns	***	***	***	**	***
QS	=		***	***	***	*	**
BE	>	>		*	*	***	***
BV	>	>	<		***	***	***
QE	>	>	>	>		***	***
TL-P	>	>	<	<	<		***
TL-T	>	>	<	<	<	>	

*bla*VIM-1

	NS	QS	BE	BV	QE	TL-P	TL-T
NS		*	***	***	***	**	**
QS	>		***	***	***	ns	***
BE	>	<		ns	**	**	***
BV	>	>	=		*	***	***
QE	>	>	>	>		***	***
TL-P	>	=	<	<	<		**
TL-T	>	>	<	<	<	>	

Spiked stool matrix

*bla*KPC-2

	NS	QS	BE	BV	QE	TL-P	TL-T
NS		*	ns	**	ns	**	
QS	<		**	**	*	ns	
BE	=	>		*	ns	*	
BV	>	>	>		***	***	
QE	=	>	=	<		***	
TL-P	<	=	<	<	<		
TL-T							

*bla*VIM-1

	NS	QS	BE	BV	QE	TL-P	TL-T
NS			*	ns		ns	
QS							
BE	>			ns		*	
BV	=		=			ns	
QE							
TL-P	=		<	=			
TL-T							

bla: beta-lactamase, NDM: New Delhi metallo-β-lactamase, OXA: oxacillinase β-lactamase, KPC: *K. pneumoniae* carbapenemase, VIM: verona integron-encoded metallo-β-lactamase, NRZ: National Reference Centre for multidrug-resistant Gram-negative bacteria, Germany
